# An Experimental Study on Processing TC4 with Nano Particle Surfactant Mixed Micro EDM

**DOI:** 10.3390/ma14206074

**Published:** 2021-10-14

**Authors:** Tingting Ni, Qingyu Liu, Zhiheng Chen, Dongsheng Jiang, Shufeng Sun

**Affiliations:** 1 Key Laboratory of Laser Green Intelligent Manufacturing Technology, Qingdao University of Technology, Qingdao 266520, China; nitingting@qut.edu.cn (T.N.); jiangdongsheng@foxmail.com (D.J.); sunshufeng@qut.edu.cn (S.S.); 2 Qingdao Haijian Energy Conservation and Environmental Protection Co., Ltd., Qingdao 266235, China; 3 Dong Fang International Container (Qingdao) Co., Ltd., Qingdao 266500, China; zhiheng1347@126.com

**Keywords:** machining performances, nano particle, micro EDM, titanium, surface surfactant

## Abstract

Micro electrical discharge machining (micro EDM) is able to remove conductive material by non-contact instantaneous high temperature, which is more suitable for machining titanium and its alloys compared with traditional machining methods. To further improve the machining efficiency and machined surface quality of micro EDM, the nano particle surfactant mixed micro EDM method is put forward in this paper. Experiments were conducted to explore the effect of nano particle surfactant on the micro EDM performance of titanium alloy. The results show that the material removal rate of micro EDM in dielectric mixed with TiO_2_ is the highest when open-circuit voltage is 100 V, followed by Al_2_O_3_ and ZrO_2_. Lower tool wear rate can be produced by using dielectric mixed with nano particle surfactant. The taper ratio of micro EDM in dielectric mixed with nano particle surfactant is higher than that in deionized water. The surface roughness Ra of micro EDM in dielectric mixed with TiO_2_ can be 50% lower than that in deionized water. It is helpful to improve the machining performance by adding surface surfactant in the dielectric of micro EDM.

## 1. Introduction

Titanium and its alloys have been widely used in aerospace and biomedical applications due to attractive properties, such as high specific strength, excellent corrosion resistance, and cryogenic properties [1,2,3]. However, as a difficult-to-machine material, titanium and its alloys are difficult to be machined by traditional machining methods, especially when involving a micro-scale feature. Micro electrical discharge machining (micro EDM) is able to remove conductive material by non-contact instantaneous high temperature, regardless of the mechanical properties of the machined materials. Therefore, micro EDM is suitable for machining difficult-to-machine material, including titanium and its alloys [4,5,6].

Although micro EDM has been widely used in aeroengine manufacturing, its application and development are seriously restricted by its low efficiency and poor surface quality. Improving the machining efficiency of micro EDM is often at the cost of sacrificing the machined surface quality, resulting in the machined surface being too rough to meet the surface quality requirements of key structures of aeroengineering [7,8,9]. In order to further improve the machining efficiency on the premise of ensuring the machined surface quality of micro EDM, particle mixed electrical discharge machining (PMEDM) technology has been studied by lots of scholars [10,11,12]. It has been indicated that the discharge state during EDM can be changed by adding Al, Si, and other micro powders into the liquid dielectric, thus, the machining efficiency is improved and the surface roughness is reduced. As a result, the contradiction between the machining efficiency and the machined surface quality is effectively alleviated [13,14].

Although the advantages of PMEDM have been reported in lots of research, the high cost and the uncertainty of the service life of powder mixed in liquid dielectric leads to the slow progress of its application in industry [15,16]. In recent years, many experts and scholars have constantly put forward corresponding new methods or theories to improve the machining efficiency and machined surface quality of PMEDM. A.P. Tiwary et al. [17] carried out experimental research on the effect of deionized water dielectric mixed with various conducting powders on various responses. They indicated that the geometrically more accurate micro-through holes on Ti-6Al-4V can be obtained by using the water dielectric mixed with various conducting powders. Y. Kim et al. [18] studied the effect of graphite powder on EDM performance, they found that the machining time of powder-mixed micro EDM drilling can be reduced by 30.9% compared with that of micro EDM drilling without powder mixed. Y. Yu et al. [19] studied the effect of rotating gas assisted perforation electrode on the machining efficiency of powder mixed EDM of titanium. They pointed out that the use of appropriate gas flow rate (0.05 L/min) enhanced the removal of debris adsorbed on bubbles during tool electrode retraction, which significantly increased the material removal rate. The existing research results show that it is an effective way to improve the machining performance by exploring the discharge characteristic and material erosion mechanism of PMEDM, and then adjust the machining effect by improving the machining process.

However, the Marangoni effect of molten metal at the discharge point has not been considered in most of the existing research on the machining mechanism of PMEDM. Marangoni effect is a fluid movement phenomenon driven by the surface tension gradient caused by the temperature gradient [20]. Under the Marangoni effect, the molten metal flows from the center of the molten pool to the surroundings. In the molten pool, the temperature gradient will cause the surface tension gradient, which makes the molten metal flow from the high temperature zone (low surface tension) to the low temperature zone (high surface tension) [21]. The results show that the surfactants CaF_2_, ATO, NaF, Cr_3_O_2,_ and TiO_2_ can shrink the discharge channel and reduce the surface tension of molten metal, that is, enhance the Marangoni effect of molten pool at the discharge point, thus, effectively increasing the penetration of stainless steel and titanium alloy weld [22,23,24]. Although there has been a lot of research on the effect of surfactant on molten metal flow in the field of welding, and surfactant has been widely used to improve welding quality, there are few relevant reports in the field on EDM [25]. It has been found that the thermocapillary forces could modify significantly the geometry of the melt pool of a single discharge, to limit the evaporation of steel at the pool surface, and to induce convection with velocities exceeding 10 m/s [26]. B. Shao et al. [27] proposed a micro-EDM model that couples the heat transfer and the fluid flow equations, and indicted that Marangoni convection is considered as the main effect on the EDM melt pool surface. M.A. Ilani et al. [28] carried out an experimental study of surfactant effects on intermolecular forces (IMF) in powder-mixed EDM of Ti-6Al-4V, they found that the fine finishing surface can be improved by 217.7% when surfactant is added. However, the effect of nano particle surfactant on the micro EDM performance is still unclear, which needs further study.

In addition, there are few studies on nano-powder mixed EDM performance [29], and the effect of nano particle surfactant ZrO_2_ on micro EDM performance has not been reported. In this paper, micro EDM experiments were conducted to explore the effect of nano particle surfactant (TiO_2_, Al_2_O_3_, and ZrO_2_) on the machining performance of titanium alloy. The evolutions of the different kinds of surfactants on the material removal rate (MRR), tool wear rate (TWR), and taper ratio (TR) were illustrated. The effect of surfactant on the surface roughness and micro morphology of machined workpiece surface were discussed.

## 2. Materials and Methods

**Figure 1:**
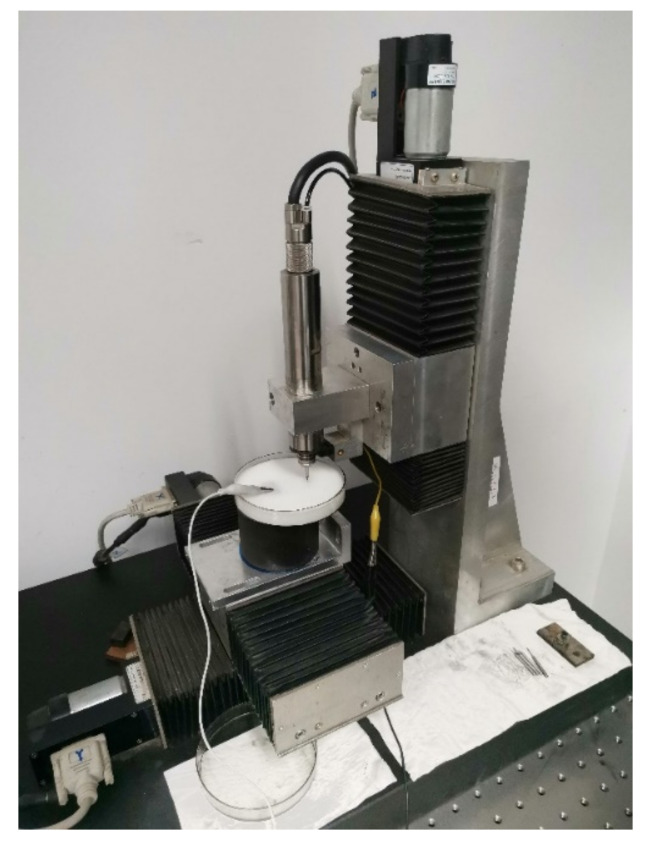
Picture of micro EDM machine tool.

The effects of nano particle surfactant on micro EDM performance were studied by conducting experiments using deionized water added with various nano particle surfactants as dielectric. The experiments were carried out based on the precision micro EDM machine independently developed by our team, as show in Figure 1. The servo feed system adopts a precise linear servo motor controlled by C863 controller produced by FAULHABER MICROMO LLC (Clearwater, USA). The travel distance of x-, y-, and z- axis is 100 mm, and the motion precision is 0.2 µm. Spindle was rotating to ensure a stable machining state, the rotational speed was kept at 2000 rpm. The workpiece was positive, while the tool electrode was negative. A resistor–capacitor circuit (RC circuit) was used to supply power for the micro EDM, the current limiting resistor was 500 Ω, and the capacitor was 1000 pF. The open-circuit voltage used in the experiments were 70, 80, 90, 100, and 110 V. Deionized water mixed with nano particles TiO_2_, Al_2_O_3_, or ZrO_2_ was used as dielectric. The nano particles were purchased from Zhuozhou Baize Trading Co., Ltd., Zhuozhou, China, and the thermo-physical properties of the nano particles are listed in Table 1. The concentration of the powder in the deionized water is 0.15 g/L, which is the same for each of them. Titanium alloy TC4 with thickness of 0.3 and 4 mm was adopted as the workpiece. The chemical composition of the workpiece material is shown in Table 2. A tungsten rod with diameter of 0.5 mm was adopted as the tool electrode for micro EDM drilling and milling. The feed depth of micro EDM drilling was 0.9 mm, and the milling length of micro EDM was 1.5 mm.

The surface roughness of the machined surface was measured by KEYENCE laser confocal microscope (VK-H2J100, KEYENCE Co., Ltd., Shanghai, China). The surface topography of the machined surface was observed using scanning electron microscope (SEM, ULTRA PLUS, Zeiss, Jena, Germany). The elemental composition of the machined workpiece surface was detected by energy dispersive X-ray (EDX, ULTRA PLUS, Zeiss, Jena, Germany). To ensure the repeatability, each test was conducted at least three times, and the average value was adopted as the result.

## 3. Results and Discussions

### 3.1. The Effect of Nano Particle Surfactant on MRR and TWR

**Figure 2:**
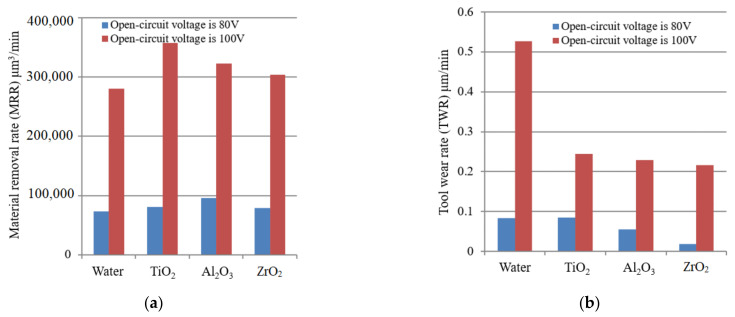
The evolution of MRR and TWR of micro EDM in various dielectric. (**a**) MRR, (**b**) TWR.

The evolution of MRR of micro EDM in dielectric mixed with different kinds of nano particle surfactant is shown in Figure 2a.

The hole drilled by micro EDM can be regarded as a circular truncated cone, therefore, the material removal volume can be calculated by the machining depth, the diameter of the entrance, and exit of the micro-hole. The volume of material removal can be obtained by the volume formula of circular truncated cone:

(1) $$V_{H}\;=\;\frac{{\pi H}_{d}}{12}\left(D_{1}^{2}+D_{1}D_{2}+D_{2}^{2}\right)$$

where, $V_{H}$ is the material removal volume of the micro hole, D_1_ is the entrance diameter of the micro hole, D_2_ is the exit diameter of the micro hole, and $H_{d}$ is the depth of the micro hole.

Thus, the MRR can be obtained from Equation (1):

(2) $$\mathrm{MRR}=\frac{V_{H}}{t_{0}}$$

where, t_0_ is the machining time of the micro hole.

It can be found that the MRR of micro EDM in dielectric mixed with nano particle surfactant is higher than that in pure deionized water, regardless of open circuit voltage. This is because the discharge gap increases in the process of micro EDM in dielectric mixed with nano particle surfactant, thus, the discharge condition is improved compared with that in the pure dielectric. As a result, the effective discharge probability increases and the discharge is more stable, causing the increase of MRR. In addition, the effect of Marangoni forces cannot be neglected in the EDM process as the calculated ratios between Marangoni force and buoyancy as well as between Marangoni and electromagnetic force are indeed both of the order of 1000 in a steady state [26]. The Marangoni effect is due to a shear stress acting on the surface of a liquid submitted to a gradient of the surface tension. It can be considered as a weak form boundary term with the equation [26]:

(3) $$\frac{\partial \gamma }{\partial T}\frac{\partial T}{\partial r}=-\eta \frac{\partial v}{\partial z}$$

where, T is the temperature, γ is the surface tension, and η is the viscosity.

In pure single-element liquids, ∂γ/∂T is negative and can be well approximated with a constant. Thus, a temperature gradient leads to forces acting from the hot (smaller γ) to the cold (larger γ) zone of the surface of a melt pool caused by a discharge. The presence of surfactant can modify the surface tension γ(x,T), which can strongly enhance the erosion rate of EDM [25]. Therefore, the MRR of micro EDM becomes higher when nano particle surfactant is mixed in the dielectric. From Figure 2a, it can also be found that the MRR of micro EDM in dielectric mixed with TiO_2_ is the highest when open-circuit voltage is 100 V, followed by Al_2_O_3_ and ZrO_2_. The MRR of micro EDM in dielectric mixed with TiO_2_ is 27.5% higher than that in the dielectric without nano particle.

The evolution of TWR of micro EDM in dielectric mixed with different kinds of nano particle surfactant is shown in Figure 2b. The electrode wear length was measured by electric contact method, making the tool contact a reference point on the workpiece surface before and after drilling each hole, and the difference of Z-axis coordinate was read as tool wear length. Thus, the TWR can be obtained by the following expression:

(4) $$\mathrm{TWR}=\frac{{\pi R}_{t}^{2}h_{1}}{t_{0}}$$

where, R_t_ is the diameter of the tool electrode, h_1_ is the electrode wear length.

It can be found that the TWR of micro EDM in dielectric mixed with nano particle surfactant is lower than that in deionized water, especially when open circuit voltage is larger. During the process of micro EDM in dielectric mixed with nano particle surfactant, the tool electrode is covered by the nano particle powder, which can protect the tool from being worn. In addition, the discharge condition of micro EDM in dielectric mixed with nano particles becomes more stable due to the larger discharge gap [30]. Therefore, both the number of abnormal discharge (short circuit, second discharge, etc.) and machining time are reduced owing to the more stable discharge state, leading to the decrease of tool wear. As a result, lower TWR can be produced by using dielectric mixed with nano particle surfactant.

### 3.2. The Effect of Nano Particle Surfactant on TR

**Figure 3:**
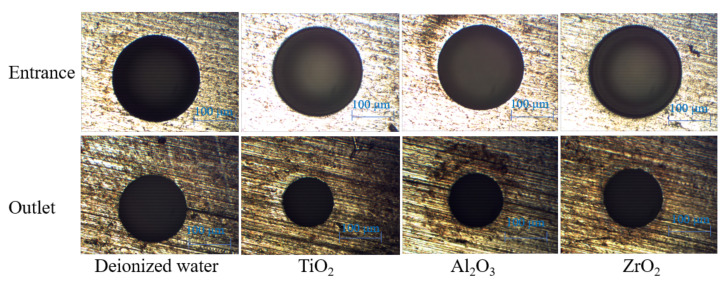
The images of the entrance and outlet of the micro holes machined in different dielectric.

The images of the entrance and outlet of the micro holes machined in different dielectric are shown in Figure 3. During the process of micro EDM drilling, the entrance of the hole endures more secondary discharges and side discharges compared with the outlet of the hole. Therefore, the hole machined by micro EDM is inevitably tapered, and the larger the aspect ratio, the larger the TR.

**Figure 4:**
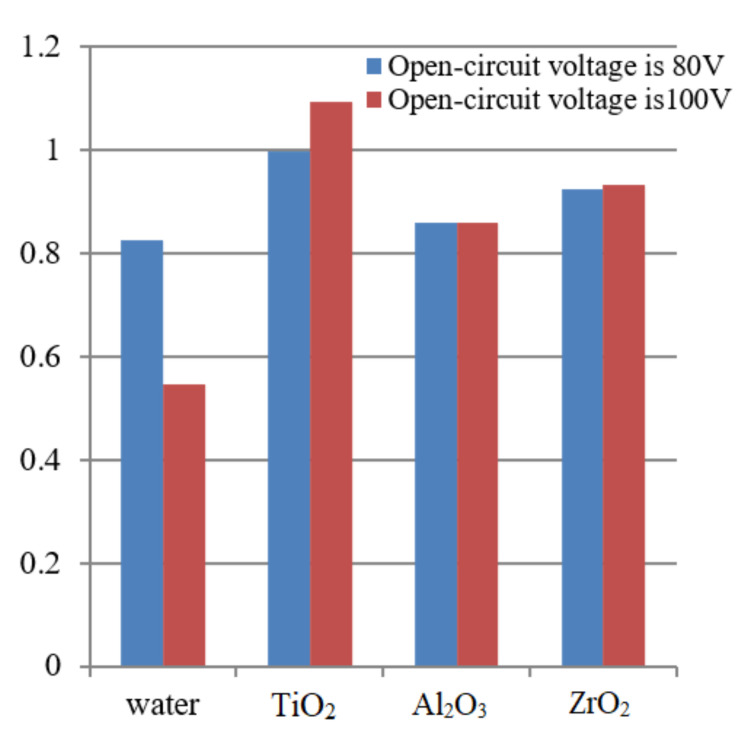
The evolution of TR of micro EDM in various dielectric.

The evolution of TR of micro EDM in dielectric mixed with different kinds of nano particle surfactants is shown in Figure 4.

The TR of the machined hole can be expressed as:

(5) $$\mathrm{TR}\;=\;\frac{D_{1}-D_{2}}{2H_{d}}$$

It can be seen from Figure 4 that the TR of micro EDM in dielectric mixed with nano particle surfactant is significantly higher than that in deionized water. As the discharge gap is enlarged due to the dielectric mixed with nano particle, the taper trend is strengthened. As a result, the TR of micro EDM in dielectric mixed with nano particle surfactant is higher than that in deionized water. It can also be seen from Figure 4 that the TR of micro EDM in dielectric mixed with TiO_2_ is the highest, followed by ZrO_2_ and Al_2_O_3_.

### 3.3. The Effect of Nano Particle Surfactant on Surface Topography

**Figure 5:**
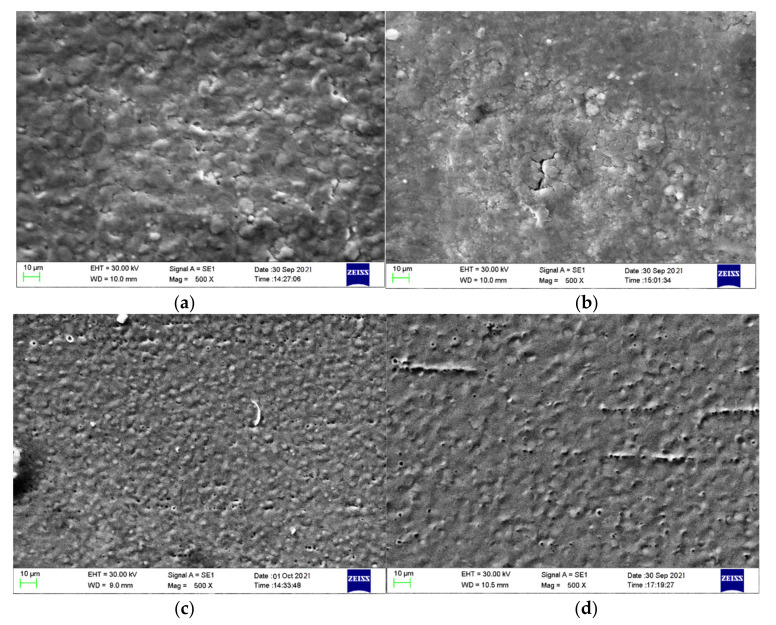
The SEM photographs of the surface machined in dielectric mixed with nano particle surfactants of (**a**) deionized water, (**b**) TiO_2_, (**c**) ZrO_2_, and (**d**) Al_2_O_3_.

The surface topography of the surface machined in dielectric mixed with different nano particle surfactants is as shown in Figure 5. It can be found that the surface machined in deionized water is significantly coarser than that in dielectric mixed with nano particle surfactants. There are micro cracks and erosion particles on the machined surfaces, and the surface machined in deionized water shows electrochemical corrosion phenomenon. Therefore, the material removal of micro EDM in deionized water is dominated by both thermal erosion and electrochemical corrosion. The machined surface is quite rough owing to the excessive electrochemical corrosion.

The existence of nano particle increases the discharge gap and improves the fluid condition of dielectric, which improves the effective discharge probability and discharge condition. In addition, each particle is under the action of high electric field due to the polarization of particles. The liquid dielectric near a particle is easily vaporized and ionized and quickly ignites the liquid dielectric near the surrounding particles, so as to expand rapidly in the discharge gap. Even if one end of the discharge structure is ignited and extinguished, the discharge structures in other directions can still continue. Therefore, the discharge plasma channel in powder mixed EDM is larger than that in ordinary EDM. When the energy is sufficient, there are many contact points between the discharge channel and the workpiece surface, so the machined surface quality is better than that of ordinary EDM. Further, a radial solidification and a homogeneous and reduced ionization time could result into formation of uniform and smaller globules and hence lower surface roughness can be observed in PMEDM process [31].

The energy spectrum analysis results indicate that there is a small amount of W and corresponding nano powder particles elements (Ti, Zr, or Al) on the workpiece surface, in addition to the elements of the base material. This is because there is element migration during the micro EDM process, due to the instantaneous high pressure and high temperature. It is also found that there is a great quantity of oxygen element on the workpiece surface, indicating that oxides are produced during spark discharge and electrochemical corrosion.

**Figure 6:**
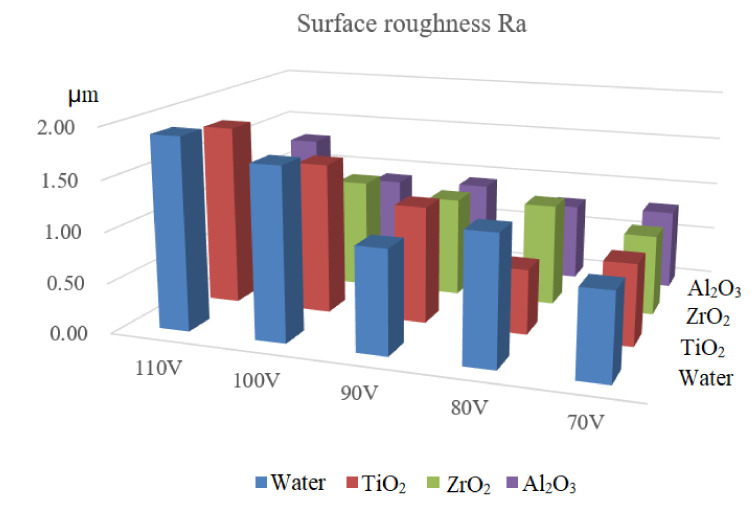
The evolution of surface roughness Ra with the type of nano particle surfactant.

The evolution of surface roughness Ra with the open-circuit voltage and different nano particle surfactants is shown in Figure 6. It can be found that the surface roughness Ra increases with the open-circuit voltage, regardless of the dielectric. The higher the open-circuit voltage, the larger the discharge energy, causing larger discharge crater and rougher machined surface. It can also be noticed that the surface roughness Ra of micro EDM in dielectric mixed with nano particle surfactant is significantly lower than that in pure deionized water, especially when the open-circuit voltage is higher. The surface roughness Ra of micro EDM in dielectric mixed with TiO_2_ can be 50% lower than that in deionized water.

## 4. Conclusions

In this paper, nano particle surfactant mixed micro EDM method is put forward to further improve the machining efficiency and machined surface quality. Micro EDM experiments were conducted to explore the effect of nano particle surfactant on the machining performance of titanium alloy. The evolutions of the different kinds of surfactants on the machining performances were analyzed and discussed. The main conclusions are drawn as followings:

- The MRR of micro EDM in dielectric mixed with TiO_2_ is the highest when open-circuit voltage is 100 V, followed by Al_2_O_3_ and ZrO_2_. The MRR of micro EDM in dielectric mixed with TiO_2_ is 27.5% higher than that in the dielectric without nano particle.
- The TWR of micro EDM in dielectric mixed with nano particle surfactant is lower than that in deionized water, especially when open circuit voltage is larger.
- The TR of micro EDM in dielectric mixed with nano particle surfactant is higher than that in deionized water, the TR of micro EDM in dielectric mixed with TiO_2_ is the highest, followed by ZrO_2_ and Al_2_O_3_.
- The surface roughness Ra of micro EDM in dielectric mixed with nano particle surfactant is significantly lower than that in deionized water, especially when the open-circuit voltage is higher. The surface roughness Ra of micro EDM in dielectric mixed with TiO_2_ can be 50% lower than that in deionized water.

## Figures and Tables

**Table 1:** The thermo-physical properties of the nano particles.

	Thermo-Physical Properties	Size/nm	Density/g·cm^−3^	Electrical Conductivity/s·cm^−1^	Thermal Conductivity/W·m^−1^K^−1^
Powder Type					
TiO_2_		10–20	4.26	<10^−10^	1.7
Al_2_O_3_		30–40	3.9	<10^−10^	7.2
ZrO_2_		30	5.89	6.0 × 10^−4^–3.3 × 10^−2^	2.09

**Table 2:** Chemical composition of Titanium alloy TC4.

Elements	Ti	Fe	C	N	H	O	Al	V
Composition (%)	Allowance	≤0.30	≤0.10	≤0.05	≤0.015	≤0.20	5.5–6.8	3.5–4.5

## Data Availability

Data is contained within the article.
